# In Vivo Dual-Modality Fluorescence and Magnetic Resonance Imaging-Guided Lymph Node Mapping with Good Biocompatibility Manganese Oxide Nanoparticles

**DOI:** 10.3390/molecules22122208

**Published:** 2017-12-12

**Authors:** Yonghua Zhan, Wenhua Zhan, Hanrui Li, Xinyi Xu, Xu Cao, Shouping Zhu, Jimin Liang, Xueli Chen

**Affiliations:** 1 Engineering Research Center of Molecular and Neuro Imaging of the Ministry of Education, School of Life Science and Technology, Xidian University, Xi’an 710071, Shaanxi, China; yhzhan@xidian.edu.cn (Y.Z.); hrli330@163.com (H.L.); xyxu@xidian.edu.cn (X.X.); caoxu327@gmail.com (X.C.); spzhu@xidian.edu.cn (S.Z.); 2 Xidian-Ningbo Information Technology Institute, Xidian University, Xi’an 710071, Shaanxi, China; 3 Department of Radiotherapy, General Hospital of Ningxia Medical University, Yinchuan 750004, Ningxia, China; zhanwhgood@163.com; 4 Key Laboratory of Biomedical Information Engineering of Education Ministry, School of Life Science and Technology, Xi’an Jiaotong University, Xi’an 710049, Shaanxi, China

**Keywords:** manganese oxide nanoparticles, fluorescence imaging, magnetic resonance imaging, lymph node, dual-modality

## Abstract

Multifunctional manganese oxide nanoparticles (NPs) with impressive enhanced *T*_1_ contrast ability show great promise in biomedical diagnosis. Herein, we developed a dual-modality imaging agent system based on polyethylene glycol (PEG)-coated manganese oxide NPs conjugated with organic dye (Cy_7.5_), which functions as a fluorescence imaging (FI) agent as well as a magnetic resonance imaging (MRI) imaging agent. The formed Mn_3_O_4_@PEG-Cy_7.5_ NPs with the size of ~10 nm exhibit good colloidal stability in different physiological media. Serial FI and MRI studies that non-invasively assessed the bio-distribution pattern and the feasibility for in vivo dual-modality imaging-guided lymph node mapping have been investigated. In addition, histological and biochemical analyses exhibited low toxicity even at a dose of 20 mg/kg in vivo. Since Mn_3_O_4_@PEG-Cy_7.5_ NPs exhibited desirable properties as imaging agents and good biocompatibility, this work offers a robust, safe, and accurate diagnostic platform based on manganese oxide NPs for tumor metastasis diagnosis.

## 1. Introduction

Sentinel lymph node (SLN) is a key parameter in clinical tumor staging and therapeutic decision-making [1,2,3]. Therefore, lymph node mapping can be used to estimate the metastatic stage of a tumor [4,5,6]. It is well known that lymph node mapping is based on lymphatic metabolism pathways, where injected bio-imaging agents are absorbed by the adjacent lymphatic system and then transported to the SLN [7,8]. Currently, the detection of SLN using vital blue dyes, raidolabeled probes, or combinations has emerged as the most common tool in the treatment of cancer patients, such as breast cancer and melanoma [9,10]. The precise detection of SLNs can impel the physicians to reduce the extent of surgical exploration, as well as postoperative morbidity [11]. Although numerous multimodal lymphatic imaging probes have been developed, several problems still remain with regard to their future clinical use; for example, low resolution, low sensitivity, and potential toxicity. Therefore, there is an urgent need to develop novel, potent clinically-translatable, multimodal lymphatic imaging probes to improve the identification rate of lymph nodes, particularly sentinel lymph nodes, in surgery.

With the emergence of molecular imaging technology [12], some kinds of imaging probes have been designed to locate SLNs in vivo, such as gold nanoparticles (NPs) [13], iron oxide [14], carbon nanotubes [15], quantum dots (QDs) [16,17], radionuclide-based NPs [18], organic dyes [19], mesoporous NPs [20], etc. According to their inherent properties, the combination of optical and MRI imaging has a big advantage over the PET/MRI, which is attributed to its very high sensitivity, low cost, non-radiative properties and ultra-high spatial resolution of magnetic resonance imaging (MRI) [12]. Therefore, the combination of optical and MRI has become attractive for both in vivo animal and clinical cancer diagnosis. To take full advantage of this hybrid optical/MRI imaging technology for future clinical applications, synthesis of novel dual-modal optical/MRI imaging probes has been actively pursued since the emergence of hardware technology in the last decade. In particular, the most representative superparamagnetic iron oxides (SPIONs) contrast agents, such as *T*_2_, have been favored for use as the MRI contrast component in optical/MRI probes over the last two decades [14,21]. However, these kinds of imaging probes have been somewhat limited in clinical applications due to their drawbacks of negative contrast and high susceptibility. Therefore, it is desirable to develop new optical/MRI probes with higher *T*_1_ or *T*_2_ enhancement to meet clinical requirements.

Recent manganese oxide NPs have been demonstrated to be one new promising *T*_1_-weighted contrast agent with high magnetization spins and fast water proton exchange rates [22,23]. Most importantly, manganese oxide NPs with good crystallinity and uniformity have been demonstrated to be easily synthesized on a large scale under mild and ambient reaction conditions [24,25]. Because of this, manganese oxide NPs, as a new class of MRI contrast agents, open a new promising direction in biomedical imaging and tumor diagnosis for future medicine [26,27]. Progress in developing multimodality imaging probes and their applications in biomedical imaging have been reported in recent years. For instance, solid and hollow MnO NPs-based *T*_1_ contrast agents have been reported for selectively imaging breast cancer cells and for drug delivery by several groups [28,29,30,31]. Yang and co-workers further developed silica-coated Mn_3_O_4_ core-shell NPs for tumor folate-receptor-targeted MRI and fluorescent imaging (FI) in vitro and tumor aptamer-receptor-targeted MRI imaging in vivo [32,33,34]. Despite many desirable properties for biomedical applications, there are currently few reports related to fusion of optical tracers and manganese oxide-based NPs into one single probe for in vivo SLN imaging. Inspired by this, we were encouraged to develop a novel optical/MRI imaging probe based on manganese oxide NPs for in vivo SLN imaging.

**Scheme 1:**
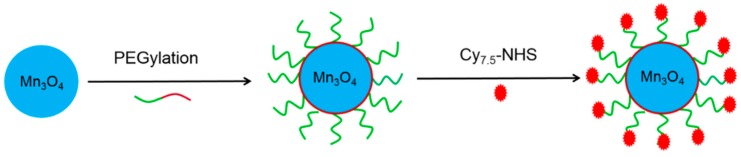
The synthetic process of Mn_3_O_4_@PEG-Cy_7.5_ nanoparticles (NPs). PEG = polyethylene glycol.

In this work, we aim to investigate in vivo SLNs mapping with surface functionalized Mn_3_O_4_ NPs. We utilized a mild and ambient reaction method to synthesize uniform Mn_3_O_4_ nanocrystals, and subsequently conjugated them with Cy_7.5_ to form Mn_3_O_4_@PEG-Cy_7.5_ NPs (PEG = polyethylene glycol) (Scheme 1). Subsequently, systematic in vivo FI/MRI imaging of the biodistribution pattern and lymph node mapping studies were carried out in normal healthy BABL/c mice to evaluate their potential capabilities as a novel dual-modality FI/MRI agent; these results were further validated by various in vitro and ex vivo experiments. Moreover, histological assessments were also carried out to determine the potential toxicity of these NPs.

## 2. Results and Discussion

### 2.1. Synthesis and Characterization of Mn_3_O_4_@PEG-Cy_7.5_ NPs

**Figure 1:**
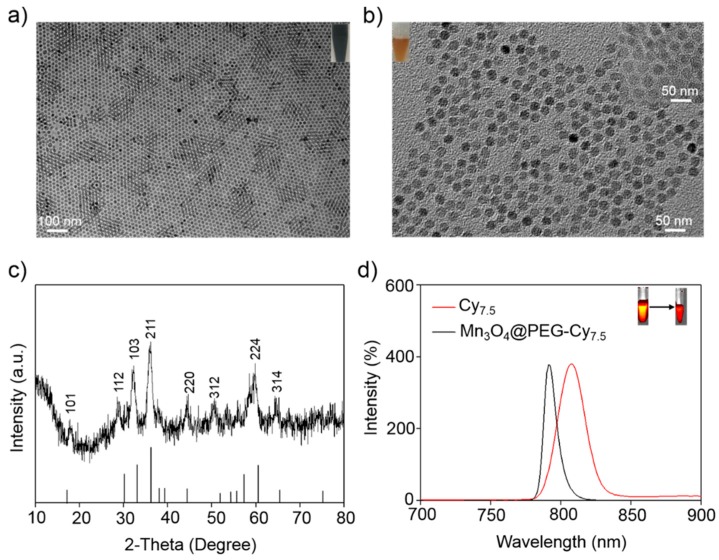
Characterization of Mn_3_O_4_@PEG NPs. (**a**) TEM images of Mn_3_O_4_ NPs; (**b**) TEM images of Mn_3_O_4_@PEG NPs and Mn_3_O_4_@PEG-Cy_7.5_ NPs (Inset); (**c**) X-ray diffraction pattern of Mn_3_O_4_ NPs; (**d**) The fluorescence imaging (FI) spectrum of Cy_7.5_ (red line) and Mn_3_O_4_@PEG-Cy_7.5_ (black line).

Various synthesis methods have been selected for preparation of the Mn_3_O_4_ NPs [24,25]. However, it is difficult to get uniform NPs to meet the desired requirements. In this work, Mn_3_O_4_ NPs were prepared according to the method reported by Hyeon [24]. As shown in Figure 1, the uniform Mn_3_O_4_ NPs were monodispersed in a nonpolar organic solvent (Figure 1a, inset) and had a small spherical shape of approximately 8 nm, which was consistent with previous reports [24]. Furthermore, the hydrophobic Mn_3_O_4_ NPs were successfully transferred to the aqueous phase by coating them with amine-functionalized PEG lipids (1,2-Distearoyl-sn-glycero-3-phosphoethanolamine (DSPE)-PEG_2000_-NH_2_) and Cy_7.5_-NHS (N-Hydroxysuccinimide) that showed good stability in an aqueous solution (Figure 1b, inset). In addition, dynamic light scattering (DLS) measurements showed that Mn_3_O_4_@PEG had a hydrodynamic diameter of 10 ± 2.3 nm, similar to the observation from TEM (Figure S1). Meanwhile, the zeta-potential value of Mn_3_O_4_@PEG (25.7 ± 4.3 mV) further verified the existence of the NH_2_ group (Figure S2). After further conjugation with Cy_7.5_-NHS, the zeta-potential values changed significantly to −10.3 ± 2.6 mV (Mn_3_O_4_@PEG-Cy_7.5_), suggesting successful conjugation of Cy_7.5_ to the surface of Mn_3_O_4_@PEG-NH_2_. Moreover, in order to further confirm the conjugation, the absorption spectra of the Cy_7.5_, M_3_O_4_, M_3_O_4_@PEG-Cy_7.5_ and DSPE-PEG-NH_2_ measured by UV spectrophotometer was also conducted. It can be seen that the Cy_7.5_ and M_3_O_4_@PEG-Cy_7.5_ NPs have obvious absorption spectra at 790 nm (Figure S3), which was ascribed to the characteristics of Cy_7.5_. Figure 1c shows the XRD pattern of the Mn_3_O_4_ NPs. Clearly, all the diffraction peaks of the as-prepared Mn_3_O_4_ NPs can be indexed as a tetragonal Mn_3_O_4_ phase (Joint Committee for Powder Diffraction Standards (JCPDS) card no: 24-0734) without metallic manganese or other oxide phases, indicating that the as-synthesized Mn_3_O_4_ NPs are crystalline and of high purity.

**Figure 2:**
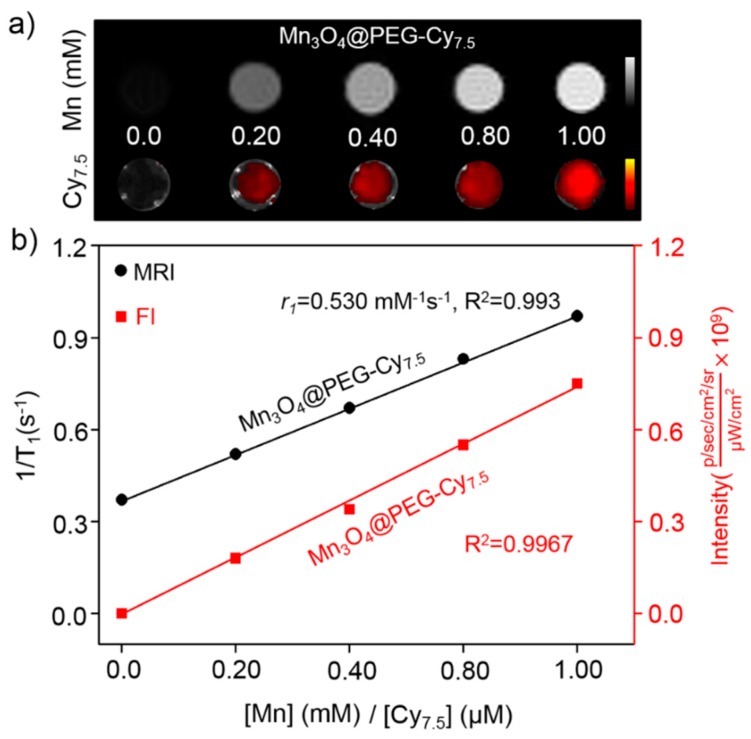
The relaxation and fluorescent properties of Mn_3_O_4_@PEG-Cy_7.5_ NPs. (**a**) *T*_1_-weighted magnetic resonance (MR) and fluorescent imaging of Mn_3_O_4_@PEG-Cy_7.5_ NPs; (**b**) *T*_1_ relaxivity plot and fluorescent intensity of aqueous suspensions of Mn_3_O_4_@PEG-Cy_7.5_ NPs on a 7 T magnetic resonance imaging (MRI) and fluorescent imaging system.

As far as we know, the optical properties of Cy_7.5_ are strongly dependent on the physical length and conformation of Cy_7.5_ chains. Figure 1d shows the fluorescent spectra behavior of Cy_7.5_ and Mn_3_O_4_@PEG-Cy_7.5_ NPs measured at the same Cy_7.5_ concentrations in dimethyl sulfoxide (DMSO). It was observed that there was a significant 18 nm blue-shift in the emission peak of the Mn_3_O_4_@PEG-Cy_7.5_ NPs compared with the Cy_7.5_ dye in DMSO. The blue-shift of the Mn_3_O_4_@PEG-Cy_7.5_ NPs can be attributed to the decreased inter-chain interactions which occur because of the conjugation of the Cy_7.5_ chain into the surface of the Mn_3_O_4_@PEG NPs [32]. These interactions allow for energy transfer from low energy to high energy which leads to the blue-shift of the Mn_3_O_4_@PEG-Cy_7.5_ NPs [35]. Although this blue shift is more attenuated by biological tissues to some extent, the peak excitation at 790 nm is still adequate to distinguish the excitation signal from the emission signal for in vivo animal imaging (Figure 1d, insect). To examine the effectiveness of the Mn_3_O_4_@PEG-Cy_7.5_ NPs as positive MRI and fluorescent contrast agents, the relaxation and fluorescent properties of Mn_3_O_4_@PEG-Cy_7.5_ NPs in aqueous media were measured by a 7 T MRI scanner and fluorescent imaging system. It was clear that Mn_3_O_4_@PEG-Cy_7.5_ NPs displayed signal enhancement in the *T*_1_-weighted magnetic resonance (MR) images and fluorescent images with increasing Mn and Cy_7.5_ concentrations (Figure 2a). The r_1_ value of the Mn_3_O_4_@PEG-Cy_7.5_ NPs was calculated from the linear fitting of the measured 1/*T*_1_ data versus Mn^2+^ concentration as 0.53 mM^−1^ s^−1^ (Figure 2b), which was in accordance with those of previously reported Mn_3_O_4_ NPs [32,33]. Although, the relaxivity of the Mn_3_O_4_@PEG-Cy_7.5_ NPs is lower than that of the commercial Gd-based agents (Gd-DTPA (diethylenetriaminepentaacetic acid), 0.41 mM^−1^ s^−1^) [22], the Mn_3_O_4_@PEG-Cy_7.5_ NPs still have their potential use value as a positive MRI and fluorescent imaging contrast agent. Moreover, maintaining stability is one of the most important factors for long-term in vivo imaging. Therefore, the stability of Mn_3_O_4_@PEG-Cy_7.5_ NPs was also investigated in phosphate-buffered saline (PBS) and 10% fetal bovine serum (FBS) at different temperatures and results are shown in Figure S4. The results indicated that the particle size of the Mn_3_O_4_@PEG-Cy_7.5_ NPs did not change significantly after two weeks in different media.

**Figure 3:**
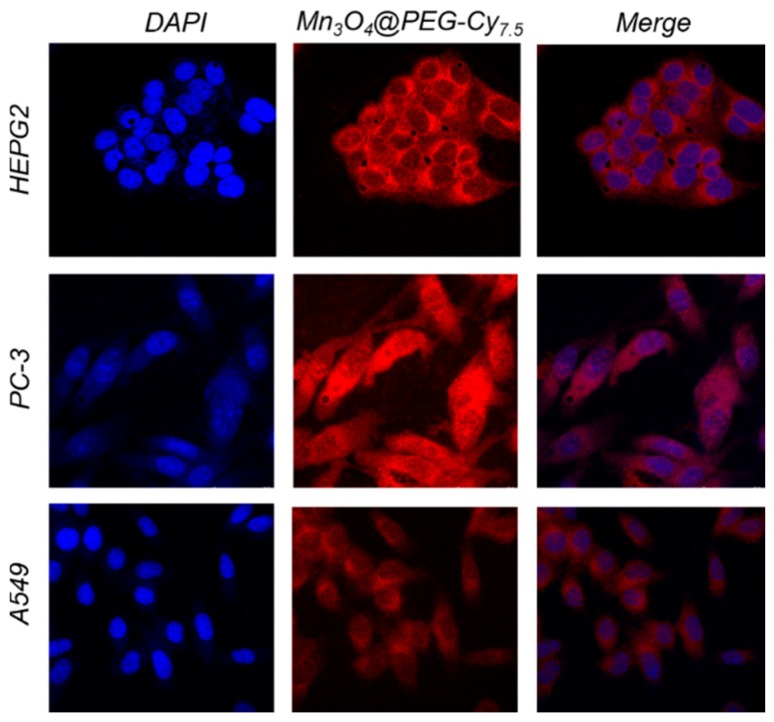
(First column) Cellular uptake of Mn_3_O_4_@PEG-Cy_7.5_ NPs in tumor cells. Mn_3_O_4_@PEG-Cy_7.5_ NPs were incubated with tumor cells for 2 h and free Mn_3_O_4_@PEG-Cy_7.5_ NPs in the medium were removed by washing. The nucleus and cytoskeleton of cells were stained and subsequently imaged by a confocal microscope. (Second column) Nuclear staining of cells with DAPI and imaging of Mn_3_O_4_@PEG-Cy_7.5_ NPs. (Third column) The merged imaging of the nucleus and Mn_3_O_4_@PEG-Cy7.5 NPs.

To further elaborate on the in vivo distribution and stability of Mn_3_O_4_@PEG-Cy_7.5_ NPs, we chose PC-3, A549, and HEPG2 cells as models to evaluate the cellular uptake ability of Mn_3_O_4_@PEG-Cy_7.5_ NPs in vitro. For this purpose, cellular uptake of NPs was verified in Figure 3. A confocal laser scanning microscopic study demonstrated that the Mn_3_O_4_@PEG-Cy_7.5_ NPs (red) were distributed in the cytoplasm and were mainly localized in the peripheral area of the nucleus. To further trace the distribution of Mn_3_O_4_@PEG-Cy_7.5_ NPs in the cells, quantitative analysis of Mn_3_O_4_@PEG-Cy_7.5_ NPs was investigated based on the detection of the content of Mn by Inductively Coupled Plasma-Atomic Emission Spectrometry (ICP-AES) at 48 h (Figure S5). After collection of the cells, they were subjected to ICP-AES analysis, the results confirmed the reasonably efficient property of Mn_3_O_4_@PEG-Cy_7.5_ NPs by cell uptake. Moreover, the Mn_3_O_4_@PEG-Cy_7.5_ NPs did not easily escape from cells and remained for a long time in the cells, suggesting that the stability of Mn_3_O_4_@PEG-Cy_7.5_ NPs in the biological environment definitely facilitates long-term in vivo imaging.

### 2.2. In Vitro Biocompatibility Studies of Mn_3_O_4_@PEG-Cy_7.5_ NPs

**Figure 4:**
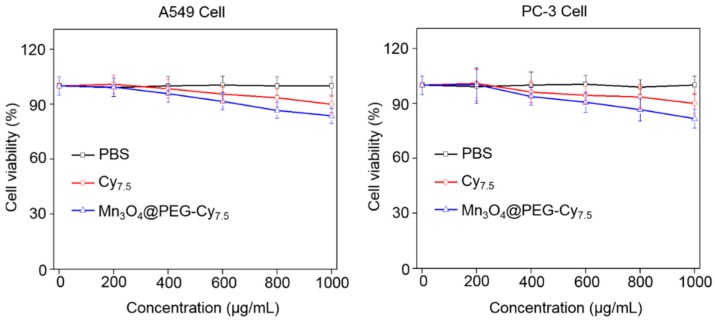
Viability of human breast cancer cells (A549) and human prostate cancer cells (PC-3) incubated with Mn_3_O_4_@PEG-Cy_7.5_ NPs at different particle concentrations for 48 h. Blue and red line indicate incubation with NPs and Cy_7.5_, while black are controls. PBS = phosphate-buffered saline.

The cytotoxicity of Mn_3_O_4_@PEG-Cy_7.5_ NPs was evaluated by Cell Counting Kit-8 (CCK-8) assay with tumor A549 and PC-3 cells. The concentration-dependent effect of Mn_3_O_4_@PEG-Cy_7.5_ NPs on the cell viability for 48 h was determined. No obvious cytotoxicity of Mn_3_O_4_@PEG-Cy_7.5_ NPs to A549 and PC-3 cells was observed at any studied concentration (from 200 to 1000 μg mL^−1^, Figure 4). Even at the concentration of 1000 μg mL^−1^, the viability for both A549 and PC-3 cells still remained above 80%, indicating that the A549 and PC-3 cells should have little cytotoxicity at the given concentration range.

### 2.3. In Vivo FI/MRI Imaging and Biodistribution Studies

**Figure 5:**
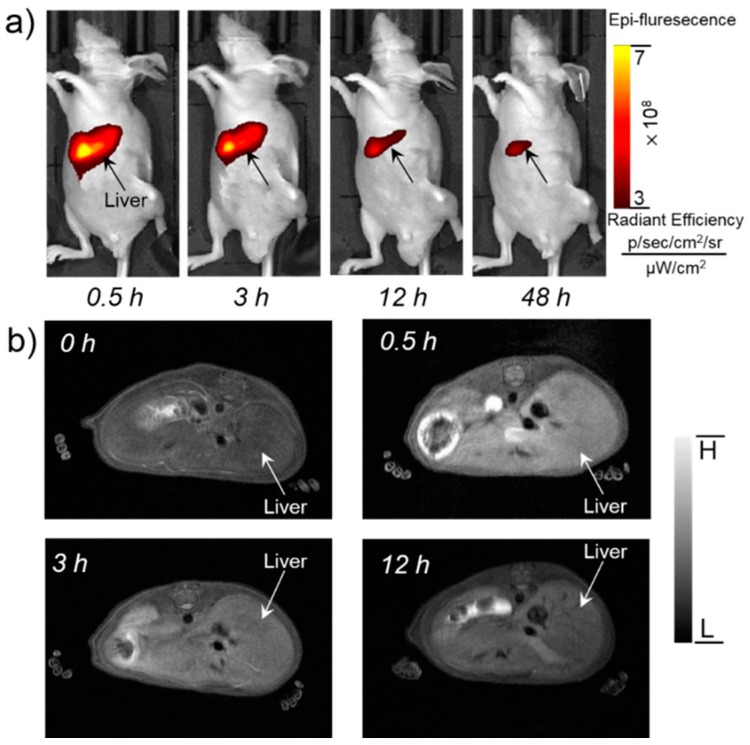
In vivo FI/MR imaging of Mn_3_O_4_@PEG-Cy_7.5_ NPs. (**a**) Serial in vivo FI imaging of Mn_3_O_4_@PEG-Cy_7.5_ NPs in BALB/c mice at different post-injection time points; (**b**) Serial in vivo MR imaging of Mn_3_O_4_@PEG-Cy_7.5_ NPs in BALB/c mice at different post-injection time points (*n* = 3 per group).

To validate the feasibility of Mn_3_O_4_@PEG-Cy_7.5_ NPs for dual-modality FI/MRI imaging and to investigate the biodistribution pattern in vivo, Mn_3_O_4_@PEG-Cy_7.5_ NPs (200 µL, 1 mg/mL) were intravenously injected into healthy BALB/c mice. Since the hydrodynamic diameters of Mn_3_O_4_@PEG-Cy_7.5_ NPs are above the cutoff for renal filtration, the route of clearance was mainly through the hepatobiliary pathway for these nanoparticles [36]. Because of this, fluorescence signals of Mn_3_O_4_@PEG-Cy_7.5_ NPs from the liver could be visualized at 0.5, 3, 12, and 48 h post-injection (p.i.) (*n* = 3). In addition, the fluorescence signals in the liver were getting weaker over time. To quantitatively and intuitively display the intensity variation, a representative region of interest (ROI) was extracted for each fluorescent image by the liver area, and the average intensity inside each ROI was calculated. The average intensity as a function of the post-injection time of the NPs is shown in Figure S4, where the red line describes the intensity variation of Mn_3_O_4_@PEG-Cy_7.5_ NPs. After imaging at 48 h, the mice were all sacrificed, and the main organs were removed to acquire the fluorescent images, as shown in Figure S6. We found that strong signals were observed in the liver and kidneys, whereas weak or even no fluorescence signals could be detected in the other main organs, which was in accordance with results in biodistribution studies (Figure 5a).

While FI provides high sensitivity and quantitative tracking of Mn_3_O_4_@PEG-Cy_7.5_ NPs, essential anatomical information is also indispensable for accurate biodistribution patterns of Mn_3_O_4_@PEG-Cy_7.5_ NPs. To further supplement the FI results, MRI with a high spatial resolution was used to investigate the Mn_3_O_4_@PEG-Cy_7.5_ NPs in BALB/c mice. As the Mn_3_O_4_@PEG-Cy_7.5_ NPs exhibited significant *T*_1_ signal enhancement in vitro, in vivo *T*_1_ MR imaging of BALB/c mice was conducted before and after intravenous injection of the Mn_3_O_4_@PEG-Cy_7.5_ NPs solution at a dose of 20 mg/kg NPs. Since the r_1_ value of the Mn_3_O_4_@PEG-Cy_7.5_ NPs was calculated as 0.53 mM^−1^ s^−1^, the injection dose was adequate for in vivo MR imaging. In view of this, a positive *T*_1_ signal enhancement in the liver was observed at 0.5 h and 3 h post-injection of Mn_3_O_4_@PEG-Cy_7.5_ NPs. However, the signal enhancement in the liver was decreased at 48 h, which could be attributed to the biodegradation and clearance of the NPs (Figure 5b). Moreover, there was no detectable signal change for the kidneys, compared with the same mice prior to the injection of the NPs. In order to further quantify the consistency between FI and MRI, by extracting the ROI using MRI compared with FI in Figure S7, the ROI of FI and MRI gradually decreased at 0.5 h, 3 h, and 12 h. This positive correlation between FI and MRI further proved the feasibility of Mn_3_O_4_@PEG-Cy_7.5_ NPs for dual-modality FI/MR imaging.

### 2.4. In Vivo FI/MRI Imaging of Lymph Nodes

**Figure 6:**
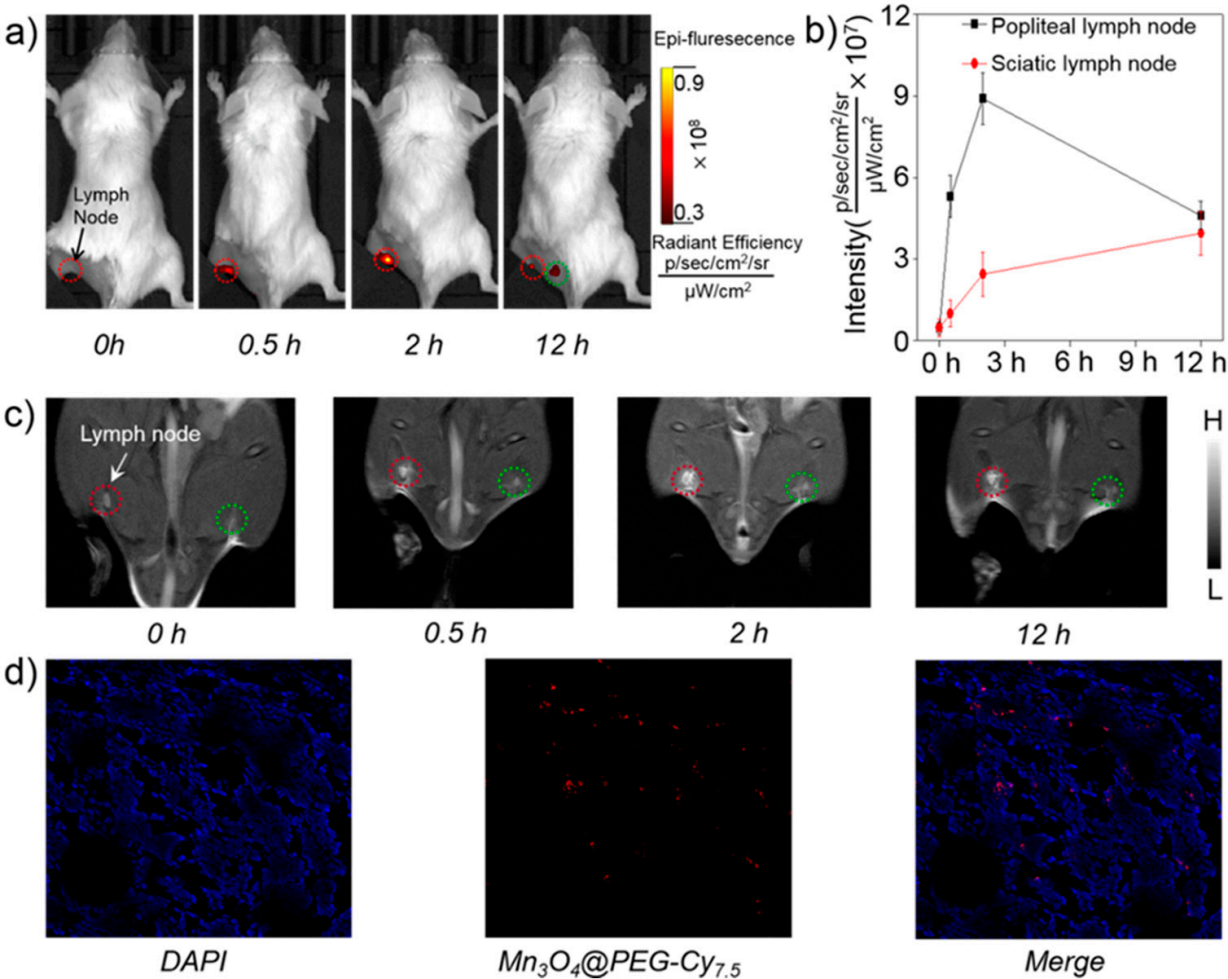
In vivo FI/MR imaging of lymph nodes with Mn_3_O_4_@PEG-Cy_7.5_ NPs. (**a**) In vivo FI imaging acquired after subcutaneous injection of Mn_3_O_4_@PEG-Cy_7.5_ NPs into the left footpad of the mouse (*n* = 3). Lymph nodes are indicated by circles; (**b**) Quantification of the Mn_3_O_4_@PEG-Cy_7.5_ NPs uptake by the lymph node (*n* = 3); (**c**) In vivo MR imaging of the lymph nodes before and after injection of Mn_3_O_4_@PEG-Cy_7.5_ NPs into the left footpad of the mouse (*n* = 3). Lymph nodes are indicated by circles; (**d**) Laser confocal images of the lymph node in mouse injected with Mn_3_O_4_@PEG-Cy_7.5_ NPs.

The lymphatic system plays a vital role in resisting disease invasion, and it is also a common site for tumor metastasis [3]. Therefore, precise identification of sentinel lymph nodes is of vital importance in both the prediction of cancer metastasis as well as determination of treatment options [7]. Considering Mn_3_O_4_@PEG-Cy_7.5_ NPs as good dual-modality FI/MRI agents in the biodistribution pattern in vivo, as a proof-of-concept, Mn_3_O_4_@PEG-Cy_7.5_ NPs were then used as non-invasive dual-modality FI/MRI probes for lymph node mapping. After Mn_3_O_4_@PEG-Cy_7.5_ NPs were subcutaneously injected into the left foot of normal healthy BALB/c mice (60 µL, 1 mg/mL), serial FI scans were carried out. As seen from Figure 6a, accumulation of Mn_3_O_4_@PEG-Cy_7.5_ NPs in the popliteal lymph node could be clearly seen at 0.5 h, 2 h and 12 h post-injection (red circle) (*n* = 3). Among them, the fluorescence signal intensity at 2 h was stronger than the other time points. In addition, it was worth mentioning that the sciatic lymph node at 12 h could also be observed clearly (blue circle). To further quantitatively and intuitively display the intensity variation, a representative ROI was extracted for each fluorescent image by the lymph node area, and the average intensity inside each ROI was calculated in Figure 6b, where the black and red line describe the intensity variation of Mn_3_O_4_@PEG-Cy_7.5_ NPs accumulated in popliteal and sciatic lymph nodes, respectively. In some ways, the above results further revealed that the accumulation of Mn_3_O_4_@PEG-Cy_7.5_ NPs in lymph nodes after injection was primarily due to the small size, which is well suited for uptake by the lymphatics.

Moreover, to further validate the accumulation of Mn_3_O_4_@PEG-Cy_7.5_ NPs in the lymph node, the nodes could be visualized by MRI, which showed gradual and prominent brightening of the popliteal lymph node after injection of Mn_3_O_4_@PEG-Cy_7.5_ NPs at 2 h and then a decrease at 12 h. Considering that the sciatic lymph node has a fluorescence signal at 12 h, MR imaging of the sciatic lymph node was also conducted. However, there was no obvious *T*_1_ contrast enhancement at the same time, which was possibly attributed to the low concentration of Mn accumulated in the sciatic lymph node. As an internal contrast, the contralateral lymph node (Figure 6c, green circle) exhibited no obvious *T*_1_ contrast enhancement at all of the same time points observed. To prove the consistency between FI and MRI, we extracted the ROIs using MRI and FI (Figure S8); both of them gradually increased at 0.5 h and 2 h, and then decreased at 12 h. This positive correlation between FI and MRI further proved the feasibility of Mn_3_O_4_@PEG-Cy_7.5_ NPs for dual-modality FI/MRI of lymph nodes. Moreover, once the lymph node was located by FI/MRI images, the area of the lymph node was submitted for a histological analysis. An unevenly aggregated distribution of the fluorescence signal was evidently observed by microscope in tissue sections from the popliteal lymph node, which indicating that the lymph node uptake of Mn_3_O_4_@PEG-Cy_7.5_ NPs was adapted to the detection of tumor metastasis to some extent, implying Mn_3_O_4_@PEG-Cy_7.5_ NPs offer a huge potential for future cancer patient diagnosis. Meanwhile, through further conjugation with other specific targeting ligands, Mn_3_O_4_@PEG-Cy_7.5_ NPs could be simultaneously improved to enable accurate early-diagnosis and targeted therapy of cancers for future clinical translation.

### 2.5. In Vivo Biocompatibility Studies of Mn_3_O_4_@PEG-Cy_7.5_ NPs

**Figure 7:**
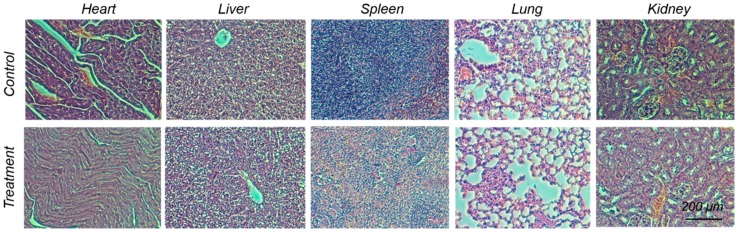
In vivo biocompatibility studies of Mn_3_O_4_@PEG-Cy_7.5_ NPs in healthy mice. Hematoxylin and eosin (H&E) staining of major organs from mice after injecting them with Mn_3_O_4_@PEG-Cy_7.5_ NPs (dose: 20 mg/kg) at 14 d post-injection. Healthy mice treated with PBS were used as the controls (*n* = 3).

To investigate the potential in vivo toxicity of Mn_3_O_4_@PEG-Cy_7.5_ NPs, histological assessment was carried out by injecting Mn_3_O_4_@PEG-Cy_7.5_ NPs (20 mg/kg) into healthy BALB/c mice via the tail vein. PBS injections served as the control. Two weeks after the injection, mice were sacrificed and major organs (heart, liver, spleen, lungs, and kidneys) were sliced and stained by hematoxylin and eosin (H&E) for a histological analysis. As shown in Figure 7, no noticeable tissue/cellular damage was observed in all major organs, as compared to that obtained from the control group. For further quantitative evaluation, serum biochemistry assays were then conducted to investigate the influence of Mn_3_O_4_@PEG-Cy_7.5_ NPs especially on potential hepatic injury and kidney functions (Table S1). Analysis of four primary hepatic function indicators, including aspartate aminotransferase (AST), alanine aminotransferase (ALT), alkaline phosphatase (ALP), and total bilirubin (TBIL), as well as two kidney function indicators, including serum creatinine (CREA) and serum urea (UREA), demonstrated no obvious hepatic or kidney disorders in both the mice treated with Mn_3_O_4_@PEG-Cy_7.5_ NPs and the control injected with PBS on day 14 post-injection. These results suggest that Mn_3_O_4_@PEG-Cy_7.5_ NPs demonstrated no obvious toxicity in mice and may be a safe agent for biomedical imaging.

## 3. Materials and Methods

### 3.1. Materials

Oleylamine (technical grade 90%), oleic acid (technical grade 90%), xylene (98%), manganese (II) acetate (98%), Cy7.5-NHS Ester, and CCK-8 were all purchased from Sigma-Aldrich. DSPE-PEG_2000_-NH_2_ was purchased from Creative PEGworks (Winston Salem, NC, USA). Prepacked Disposable Desalting Columns-10 (PD-10) were acquired from GE Healthcare. All buffers and water were Millipore grade. All chemicals were used as received without further purification.

### 3.2. Characterization

The size and morphology of Mn_3_O_4_ NPs were observed using an FEI T12 transmission electron microscope (TEM) operated at an accelerating voltage of 120 kV. X-ray diffraction (XRD) measurements were performed on a Bruker D8 diffractometer with Cu Ka radiation (λ = 0.15405 nm). The surface zeta potential and hydrodynamic size were measured using a Malvern Zetasizer Nano ZS. The *T*_1_-relaxivities were measured in a 7 T magnet, and *T*_1_-weighted images were acquired with a conventional spin echo acquisition (repetition time, TR, 1000 ms) with echo time, TE, of 50 ms, and a section thickness of 1 mm in a 7 T small animal scanner (Bruker, Karlsruhe, Germany). Relaxivity values of *r*_1_ were calculated through curve fitting of 1/*T*_1_ relaxation time (s^−1^) versus the Mn concentration (mM). The fluorescence spectra and intensity were recorded using the IVIS imaging system (IVIS Lumina Series III, PerkinElmer, Fremont, CA, USA).

### 3.3. Synthesis of the Mn_3_O_4_@PEG-Cy_7.5_ NPs

Mn_3_O_4_ NPs were prepared according to a previously reported method with slight modifications [24]. Manganese (II) acetate (1 mmol, 0.17 g) and a mixture of oleic acid (2 mmol, 0.57 g) and oleylamine (10 mmol, 2.67 g) were dissolved in 15 mL of xylene in air atmosphere. After slowly heating to 95 °C, 1 mL of deionized (DI) water was injected into the solution under brisk stirring, and the resulting solution was aged at 95 °C for 3 h. A total of 100 mL of ethanol was then added to precipitate the nanocrystals, followed by centrifugation to retrieve the nanocrystals in powder form. Ten milligrams of Mn_3_O_4_ nanocrystals were dispersed in 1 mL of chloroform. Then, 20 mg of DSPE-PEG_2000_-NH_2_ in 2 mL chloroform were added to the mixture and ultrasonicated for 2 min. After evaporating the solvent by argon blowing, the residue was incubated at 60 °C in a vacuum for 1 h. Upon the addition of 15 mL of water, a transparent brown solution was generated. After filtration (0.22 μm syringe filter, cellulose acetate), excess DSPE-PEG_2000_-NH_2_ was removed by ultracentrifugation (40,000 rpm, 1 h, 2 times) and Mn_3_O_4_@PEG NPs were obtained. About 1 mg Cy7.5-NHS was dissolved into 200 μL of DMSO to make the final concentration of 5 mg/mL, and then 6 μL of the Cy7.5-NHS solution was added into the Mn_3_O_4_@PEG NPs solution. After mixing and reacting for 2 h at room temperature, the Mn_3_O_4_@PEG-Cy_7.5_ NPs were obtained. To ensure that Mn_3_O_4_@PEG-Cy_7.5_ NPs were sufficiently stable for in vivo applications, stability studies were carried out. Mn_3_O_4_@PEG-Cy_7.5_ NPs were incubated in PBS and 10% FBS at 25 °C and 37 °C for up to two weeks, and DLS analysis was performed as previously described [33].

### 3.4. Cell Cytotoxicity Studies of Mn_3_O_4_@PEG-Cy_7.5_ NPs

The cytotoxicity of Mn_3_O_4_@PEG NPs was assessed with a CCK-8 assay using PC-3 cells and A549 cells. Briefly, cells were seeded in 96-well plates at 20,000 cells per well in 200 µL culture medium. The cells were maintained in Roswell Park Memorial Institute (RPMI)-1640 containing 10% fetal bovine serum (FBS) and incubated at 37 °C in a humidified cell culture incubator with 5% CO_2_ atmosphere for 24 h. Mn_3_O_4_@PEG-Cy_7.5_ NPs solutions with different concentrations from 200 to 1000 μg/mL were added to each well, and the cells were subjected to a CCK-8 assay after being incubated for another 24 h. The cell viability was determined by measuring the absorption at 450 nm using a microplate reader. Cell viability was calculated using: cell viability (%) = (mean absorption value of treatment group/mean absorption value of control) × 100.

### 3.5. Cellular Uptake Ability of Mn_3_O_4_@PEG-Cy_7.5_ NPs

The cellular uptake of the samples was observed by a fluorescent microscope (Eclipse Ti-S, Nikon, Japan). The cells were cultured for 48 h in 24-well flat-bottomed plates, with Mn_3_O_4_@PEG-Cy_7__.5_ coated cover glasses in each well. A confocal microscope (TCS SP5 II, Leica, Berlin, Germany) with a laser excitation at 458 and 514 nm was used in the detection. To confirm the endocytosis of the Mn_3_O_4_@PEG-Cy_7.5_, the cellular uptake behavior of the Mn_3_O_4_@PEG-Cy_7.5_ was observed using a confocal laser scanning microscope (CLSM). A549, HEPG2, and PC-3 cells attached to 6-well plates covered with cover glass were treated with Mn_3_O_4_@PEG-Cy_7.5_. After incubation in the dark for a predetermined time, the cells were rinsed twice with PBS (pH 7.4) and fixed using 4% paraformaldehyde solution, and they were then visualized using an inverted fluorescence microscope (Eclipse Ti–S, Nikon, Tokyo, Japan).

### 3.6. In Vivo Toxicity Studies of Mn_3_O_4_@PEG-Cy_7.5_ NPs

The toxicity of Mn_3_O_4_@PEG NPs in healthy male BALB/c mice was evaluated by injecting Mn_3_O_4_@PEG-Cy_7.5_ NPs (dose: 20 mg/kg) via the tail vein. Mice injected with only PBS served as a control group (*n* = 3). Mice were sacrificed to collect blood for serum biochemistry assays on day 14. At the same time, major organs from each mouse were harvested and fixed in 4% a paraformaldehyde solution for 1 day. These tissues were then embedded in paraffin and stained with hematoxylin and eosin (H&E) and examined using a digital microscope (Leica DM5000). Examined tissues included the heart, liver, spleen, lung, and kidney. The serum chemistry data, including hepatic and kidney functions, were measured by the Animal Center of Xi’an Jiaotong University (Number XJTULAC 2016-412).

### 3.7. In Vivo FI/MRI Imaging of Lymph Nodes and Biodistribution Studies

FI scans of BALB/c mice (*n* = 3 per group) at 0.5, 3, 12 and 48 h post-injection (p.i.) with 200 µL Mn_3_O_4_@PEG-Cy_7.5_ NPs were performed using the IVIS imaging system (IVIS Lumina Series III, PerkinElmer, Fremont, CA, USA) following tail vein injection. For in vivo lymph node mapping with FI, 60 µL of Mn_3_O_4_@PEG-Cy_7.5_ NPs was subcutaneously injected into the left footpad of healthy BALB/c mice. Selected times of 0.5 h, 2 h and 12 h were used for serial FI scans. In vivo *T*_1_-weighted MR imaging was performed at 0.5 h, 3 h and 12 h post-injection after intravenous injection with 200 μL Mn_3_O_4_@PEG with a Mn concentration of 1 Mm using a 7 T small animal scanner (Bruker, Karlsruhe, Germany) with the following parameters: Repetition Time (TR) = 400 ms; Echo Time (TE) = 10 ms; flip angle = 120°; Field of View (FOV) = 35 mm × 35 mm; matrix = 256 × 256; NEX = 8; slice thickness = 1 mm for axial liver images and 0.5 mm for coronal lymph node mapping. All animal studies were conducted under a protocol approved by the University of Xi’an Jiaotong Animal Care and Use Committee (Number XJTULAC 2016-412).

## 4. Conclusions

In conclusion, we developed a robust, safe, and accurate *T*_1_-MRI and FI contrast agent, Mn_3_O_4_@PEG-Cy_7.5_ NPs. Compared with other prevalent Mn_3_O_4_-based NPs reported to date, Mn_3_O_4_@PEG-Cy_7.5_ NPs is a successful utilization example for in vivo dual-modality FI and MRI-guided lymph node mapping, which is attributed to its high sensitivity of FI and high soft-tissue resolution of MRI. Since Mn_3_O_4_@PEG-Cy_7.5_ NPs exhibited desirable properties as imaging agents and good biocompatibility, this work also offers a potent, precise diagnostic platform for tumor metastasis in the future.

## Supplementary Materials

Supplementary materials are available online.
