# Supplementary material for: In Vivo Dual-Modality Fluorescence and Magnetic Resonance Imaging-Guided Lymph Node Mapping with Good Biocompatibility Manganese Oxide Nanoparticles
Source: Molecules. 2017 Dec 12;22(12):2208. doi: 10.3390/molecules22122208 (PMC6149721; doi:10.3390/molecules22122208)
Supplement: Supplementary file 1 [file molecules-22-02208-s001.pdf]

molecules

MDPI

Supporting Information

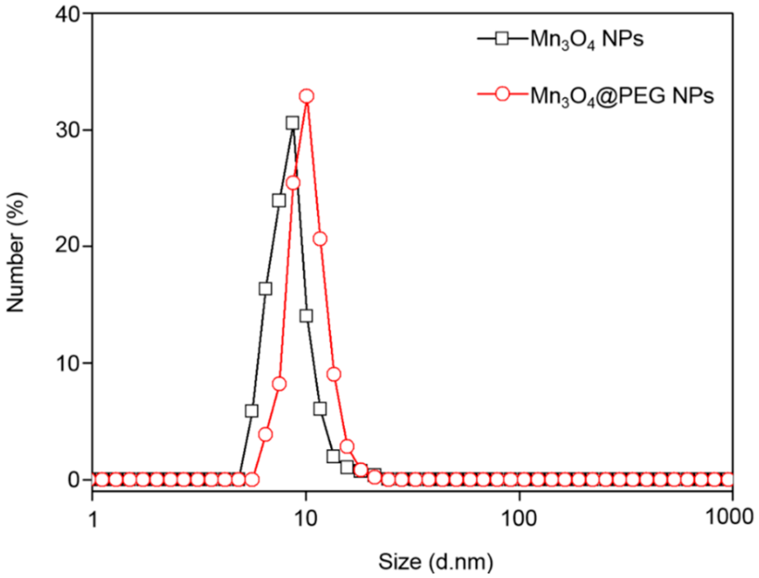

Figure S1. Size analysis of Mn_3O_4 and Mn_3O_4@PEG NPs by dynamic light scattering (DLS).

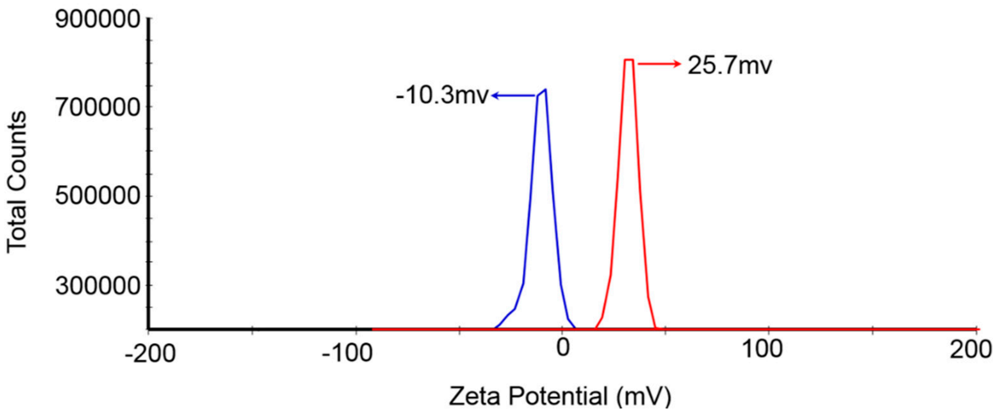

Figure S2. Zeta potential analysis of Mn_3O_4@PEG NPs and Mn_3O_4@PEG-$Cy_{7.5}$ NPs by Malvern Zetasizer Nano ZS.

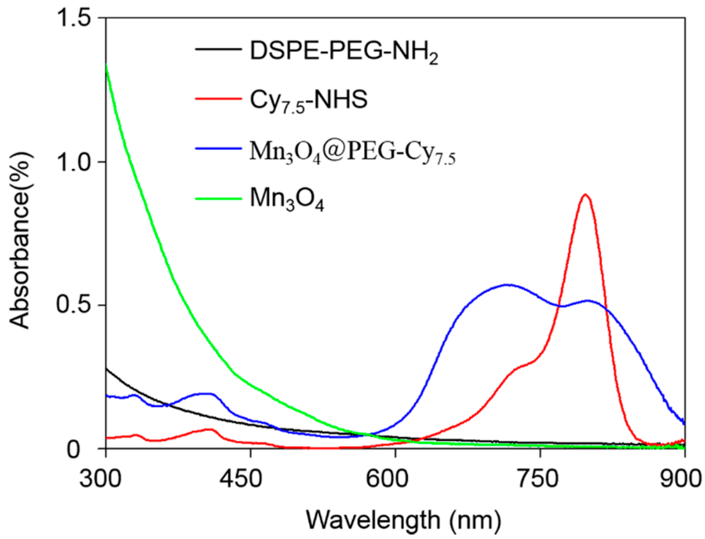

Figure S3. Absorbance spectra of Cy7.5-NHS, Mn_3O_4, Mn_3O_4@PEG-Cy7.5, and DSPE-PEG-NH_2 as determined by UV spectrophotometer. NHS = N-Hydroxysuccinimide; DSPE = 1,2-Distearoyl-sn-glycero-3-phosphoethanolamine.

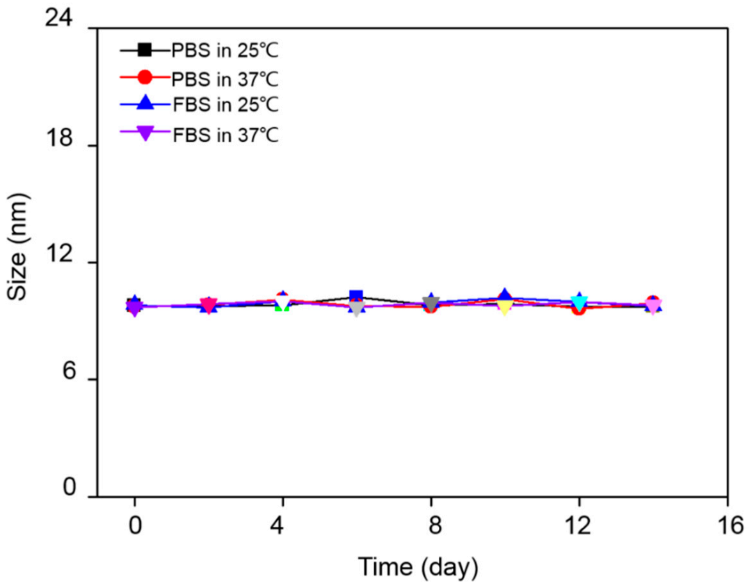

Figure S4. The size stability of Mn_3O_4@PEG-Cy7.5 NPs in PBS and 10% fetal bovine serum (FBS) varies with time and different temperatures in two weeks.

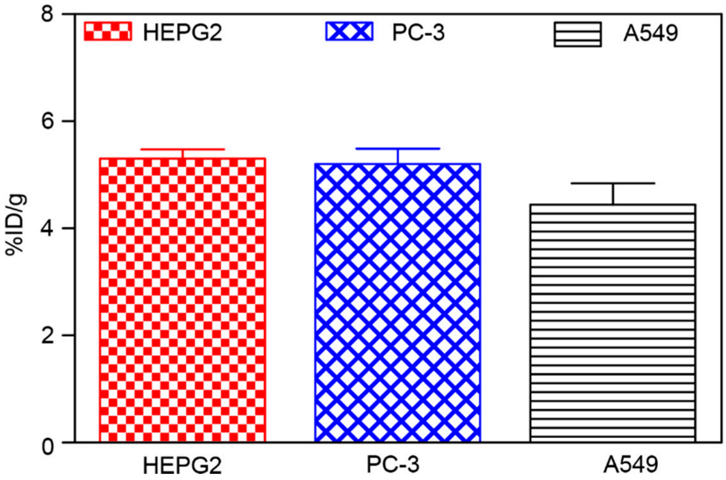

Figure S5. Quantitative analysis of cell uptake based on the detection of the content of Mn at 48 h by Inductively Coupled Plasma-Atomic Emission Spectrometry (ICP-AES) (n = 3).

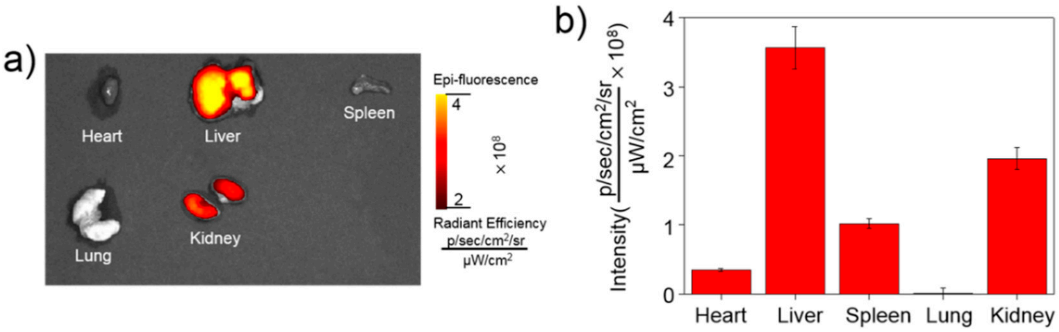

Figure S6. Ex vivo imaging of Mn_3O_4@PEG-$Cy_{7.5}$ NPs at 48 h following intravenous injection of Mn_3O_4@PEG-$Cy_{7.5}$ NPs (n = 3 per group). (a) Ex vivo FI images of main organs. (b) Quantitative analysis of the FI images of the main organs (n = 3 per group).

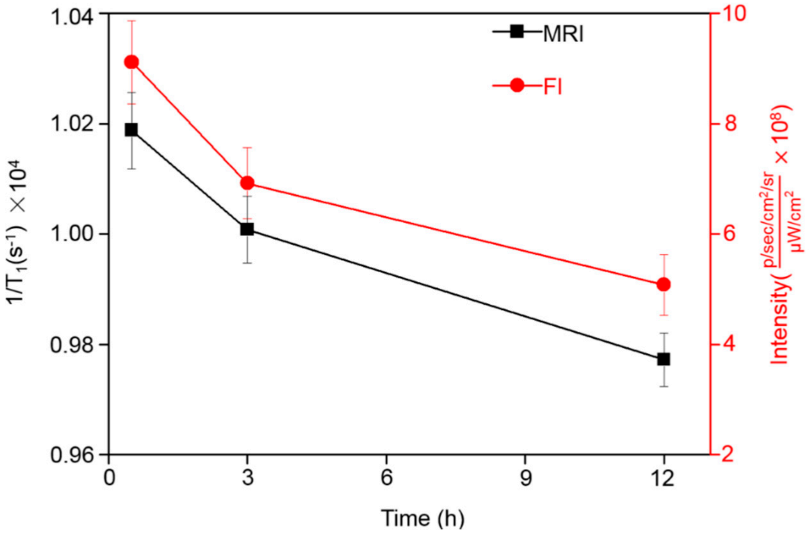

Figure S7. Quantitative analysis of the FI and MRI biodistribution pattern data in vivo. Time-activity curves of the liver following intravenous injection of Mn_3O_4@PEG-$Cy_{7.5}$ NPs at 0.5 h, 3 h, and 12 h (n = 3 per group).

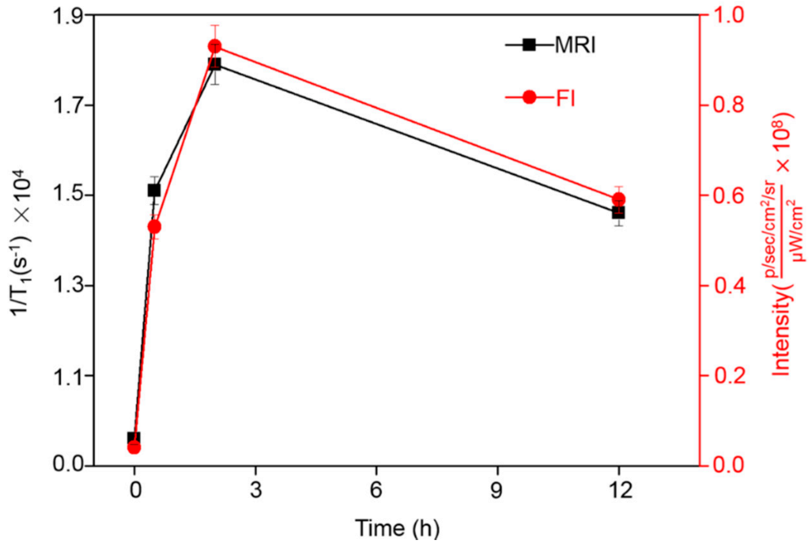

Figure S8. Quantitative analysis of the FI and MRI data in lymph nodes. Time-activity curves of the lymph node following subcutaneous intravenous injection of Mn_3O_4@PEG-$Cy_{7.5}$ NPs at 0.5 h, 2 h, and 12 h (n = 3 per group).

Table S1 Serum level of liver and kidney function markers in mice 2 weeks after injection of Mn_3O_4@PEG-$Cy_{7.5}$ NPs (20 mg/kg). n = 3. AST = aspartate aminotransferase; ALT = alanine aminotransferase; ALP = alkaline phosphatase; TBIL = total bilirubin (TBIL); CREA = serum creatinine; UREA = serum urea.

Sample	ALT (U/L)	AST (U/L)	ALP (U/L)	ADA (U/L)	UREA(mmol/L)	CREA(μmol/L)
Control	53.49 ± 2.56	133.45 ± 4.38	110.37 ± 3.15	5.6 ± 0.43	13.32 ± 0.19	2.4 ± 0.04
NPs	51.27 ± 2.38	131.37 ± 3.19	113.29 ± 4.11	5.9 ± 0.39	13.45 ± 0.17	2.1 ± 0.03
